# Gender-specific selection on codon usage in plant genomes

**DOI:** 10.1186/1471-2164-8-169

**Published:** 2007-06-13

**Authors:** Carrie-Ann Whittle, Meghna R Malik, Joan E Krochko

**Affiliations:** 1 Plant Biotechnology Institute, National Research Council of Canada, 110 Gymnasium Place, Saskatoon, SK, S7N 0W9, Canada

## Abstract

**Background:**

Currently, there is little data available regarding the role of gender-specific gene expression on synonymous codon usage (translational selection) in most organisms, and particularly plants. Using gender-specific EST libraries (with > 4000 ESTs) from *Zea mays *and *Triticum aestivum*, we assessed whether gender-specific gene expression *per se *and gender-specific gene expression level are associated with selection on codon usage.

**Results:**

We found clear evidence of a greater bias in codon usage for genes expressed in female than in male organs and gametes, based on the variation in GC content at third codon positions and the frequency of species-preferred codons. This finding holds true for both highly and for lowly expressed genes. In addition, we found that highly expressed genes have greater codon bias than lowly expressed genes for both female- and male-specific genes. Moreover, in both species, genes with female-specific expression show a greater usage of species-specific preferred codons for each of the 18 amino acids having synonymous codons. A supplemental analysis of *Brassica napus *suggests that bias in codon usage could also be higher in genes expressed in male gametophytic tissues than in heterogeneous (flower) tissues.

**Conclusion:**

This study reports gender-specific bias in codon usage in plants. The findings reported here, based on the analysis of 1 497 876 codons, are not caused either by differences in the biological functions of the genes or by differences in protein lengths, nor are they likely attributable to mutational bias. The data are best explained by gender-specific translational selection. Plausible explanations for these findings and the relevance to these and other organisms are discussed.

## Background

Although tissue-specific gene expression has been associated with bias in codon usage in certain multicellular organisms including humans, *Drosophila melanogaster*, and *Arabidopsis thaliana *[1-5], very little data currently exists for most organisms, particularly regarding the role of gender-specific tissues and gametes. One of the few studies addressing the effect of gender, in *Drosophila*, suggests that genes having a higher ratio of female to male expression have a greater bias in codon usage [4,6]. A largely unstudied biological system where gender-specific gene expression could significantly alter codon usage is plants. Recent findings, in *A. thaliana*, have indicated that male gametes pass on a greater number of induced harmful mutations to their offspring, suggesting that mutations are subject to less selection in male tissues/gametes than in female tissues/gametes [7]. Such findings at the population level (short-term), suggest that gender could also impact the selective processes that alter molecular evolution in plants, including the usage of synonymous codons. Given that gender-specific selective pressures on codon usage could alter gene evolution and structure, and thereby influence population genetics, disease, and/or reproductive biology, and given the general lack of data to date, further investigation is warranted. Here, we focus on the evaluation of gender-specific codon usage in plants.

Nonrandom use of synonymous codons is a prevalent phenomenon observed in a diverse range of organisms [1,8-17]. A bias in codon usage occurs when synonymous codons are not all used at the same frequency in coding DNA [14,18,19]. Such bias in codon usage could result from mutational pressure, as indicated by a positive correlation between the nucleotide content of third codon positions and adjacent introns [20-23], or from selective pressure. Selective pressure has been supported by two findings. Firstly, greater levels of bias in codon usage are generally associated with a greater frequency of "preferred" codons (those used most frequently in the most biased genes) [24], a trend that corresponds to the abundance and/or gene number of tRNA in bacteria, yeast, *C. elegans*, *Drosophila*, *Arabidopsis *and other organisms [1,8,9,12-15,25-27]. Secondly, bias in codon usage has been well correlated to the level of gene expression, with the greatest bias occurring in highly expressed genes [8,10,12,28,29]. Each of these findings suggests that the use of preferred codons confers fitness benefits that enhance translational efficiency, a phenomenon particularly advantageous for the highly expressed genes [8,12]. In this regard, gene expression level is an essential component of understanding gender-specific influences on codon usage.

The main challenges for comparing male and female codon usage relative to gene expression for plant species, where the availability of genomic DNA sequences is often limited, are obtaining sufficient coding DNA data to assess codon usage in those tissues and determining the level of gene expression. EST datasets provide an effective solution to both issues. In particular, EST data have proven to be an effective means of quantifying gene expression in a range of tissues as the extent of redundancy in ESTs reflects the abundance of mRNA in the tissue or cells from which the library was obtained [8,10,12,30-32]. In addition, the increased availability of EST data, the long sequence length (200 to 800 bp) and the level of accuracy of the sequence data (from efficient sequence techniques and base-calling software, and the general use of only high quality reads) [33-35] makes it possible to study codon usage directly from EST sequences, even before the corresponding genomic sequences are available [36,37]. In this regard, the recent availability of male- and female-specific EST libraries in plants provide an effective resource to better understand selection on codon usage and how it may be influenced by gender.

In the present study, the main goal was to assess whether gender-specific gene expression *per se *and gender-specific gene expression level are correlated with codon usage in *Zea mays *and *Triticum asestivum*. As a supplemental analysis, we compared the bias in codon usage for genes expressed only in gametophytic tissue (male germline cells, microspores) and in flower tissue (composed of the somatic and reproductive tissues) at both high and low expression levels in *Brassica napus*. Given that gene function and protein length [8,10], have previously been found to influence codon usage, we also evaluated the role of those parameters within our analysis.

## Results

In order to compare codon usage relative to gender-specific expression, we collected data from sperm and egg EST libraries for *Z. mays *and anther and ovary libraries for *T. aestivum *(Table 1). Microspore and flower libraries were obtained for *B. napus*. In brief, we obtained gender/tissue-specific datasets in the following manner: 1) clustering and assembly of ESTs from each library using CAP3 [38], 2) identification of unigenes (from now forward referred to as "genes") having translation products that matched known or hypothetical proteins in *A. thaliana *(using BLASTX [39-41]), 3) extraction of genes with tissue-specific expression by comparisons between the two compared tissues for each species (using MEGABLAST) [41], and 4) determination of the expression level per gene (based on the number of ESTs, see Methods). The six tissue-specific sequence datasets obtained are: *Z. mays *sperm-specific genes (*N *= 955), *Z. mays *egg-specific genes (*N *= 946), *T. aestivum *anther-specific genes (*N *= 3326), *T. aestivum *ovary-specific genes (*N *= 1489), *B. napus *microspore-specific genes (*N *= 1675) and *B. napus *flower-specific genes (*N *= 3181). Note that gender-specific genes represent those that are specific to a particular tissue or gamete (e.g. sperm) when compared to only one other tissue or gamete (e.g., egg,) and not relative to all tissues from the plant. Thus, these gene sets are larger than one would have found if the ESTs had been compared to all tissues of a plant. Subsequently, the GC content at third nucleotide positions (*GC3*) and the frequency of preferred codons (*Fpr*), each of which have been shown to be effective indicators of bias in codon usage [1,8,31], were determined for every gene from each of the tissue-specific datasets. Bias in codon usage was quantified using a single EST sequence to represent each gene (i.e., genes are represented by the longest EST in the contig or a singleton EST, see Methods). The data show, as described in detail below, that genes specific to female tissues and gametes have a greater bias in codon usage in both *Z. mays *and *T. aestivum *than genes expressed in male tissues and gametes. As well, male microspores have a greater bias than the heterogeneous tissues of the flower in *B. napus*.

**Table 1:** The cDNA libraries used in the present investigation. The number of ESTs from each library that match genes in *Arabidopsis thaliana*, and the associated number of contigs and singletons are provided.

Species and tissue or cell type	Relevant properties of tissue or cells	Name of library in NCBI and contributor(s)	Total number of ESTs per library (March 2006)	Number of ESTs matching genes in *Arabidopsis *(e < 10^-7^)	Number of contigs identified using CAP3	Number of singletons identified using CAP3	Unigenes (contigs plus singletons)
***Zea mays***							
Sperm	Male	*Zea mays *sperm cell cDNA library (S. McCormick) Engel et al.[64]	5174	2457	384	781	1165
Egg	Female	*Zea mays *egg cell cDNA library (H. Yang and S. McCormick) Yang et al. [65]	4085	1957	264	863	1127
							
***Triticum aestivum***							
Anther-meiotic	Male	Wheat meiotic anther cDNA library (O. Anderson)	9139	6118	860	3058	3918
Ovary	Female	Ov (Genoplante)	5311	3699	813	1295	2108
							
***Brassica napus ***							
Microspores (from 3 to 4 mm buds)	Male	65JKBNM0 (M. Malik and J. Krochko)	6539	5325	781	1620	2401
Buds and young flowers	Heterogeneous (male, female and somatic)	ADIS-MPIZ 021 (B. Weisshaar)	8747	6164	995	2665	3660

**Figure 1:**
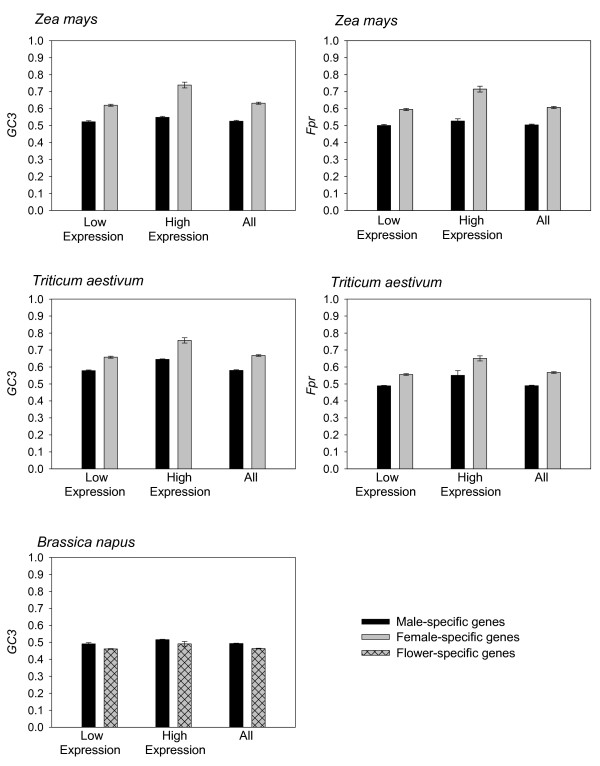
The GC content at third codon positions and the frequency of preferred codons for genes expressed specifically in male and female gametes or tissues in *Zea mays *and *Triticum aestivum *and the GC content of male-specific and flower-specific genes in *Brassica napus *relative to gene expression level. *P*-values between male and female (flower for *B. napus*) expressed genes for *GC3 *and for *Fpr *are as follows (respectively, in parenthesis):*Z. mays*: high (< 1 × 10^-16^**, < 1 × 10^-16^**), low (< 1 × 10^-16 ^**, < 1 × 10^-16^**), all expression levels (< 1 × 10^-16^**, < 1 × 10^-16^**); *T. aestivum*: high (< 1 × 10^-16^*, 0.001*), low (< 1 × 10^-16^**, < 1 × 10^-16^**), all expression levels (< 1 × 10^-16^**, < 1 × 10^-16^**); *B. napus *(*GC3 *only): high (0.141), low (< 1 × 10^-16^**) and all expression levels (< 1 × 10^-16^**). *P*-values for high versus low expression level within gender (or flower): *Z. mays*: Female (< 1 × 10^-16^**, < 1 × 10^-16^**), Male (0.012*, 0.010*); *T. aestivum*: Female (< 1 × 10^-16^**, < 1 × 10^-16^**), Male (0.036*, 0.025*); *B. napus*: Male (1 × 10^-16^**), Flower (0.018*). *P*-values for lowly expressed female-specific genes versus highly expressed male-specific genes: *Z. mays *(< 1 × 10^-16^**, < 1 × 10^-16^**) and *T. aestivum *(0.867, 0.553). *P*-values for highly expressed female-specific genes versus lowly expressed male-specific genes: *Z. mays *(< 1 × 10^-16^**, < 1 × 10^-16^**) and *T. aestivum *(< 1 × 10^-16^**, < 1 × 10^-16^**). *P*-value for highly expressed male-specific genes versus lowly expressed genes in flower in *B. napus *(0.760). *P*-value for highly expressed male-specific genes versus lowly expressed genes in the flower in *B. napus *(< 1 × 10^-16^).* Indicates statistical significance (*P *< 0.05), **Indicates that *P*-value remains statistically significant after the Bonferroni correction.

The GC content at third nucleotide positions and the frequency of preferred codons were each statistically significantly higher for genes expressed specifically in eggs as compared to sperm in *Z. mays *and for genes expressed in ovary as compared to anther in *T. aestivum *(Figure 1). This result was statistically significant for genes expressed at both high and at low levels (high > 5 ESTs per 10 000; low ≤ 5 ESTs per 10 000) as well as across all genes. Statistically significant higher values for *GC3 *were detected in microspore-specific genes as compared to the flower-specific genes in *B. napus *for genes expressed at low levels, but not for highly expressed genes (only *GC3 *was determined for *B. napus *as preferred codons have not been described yet). Within the male-specific and the female-specific genes, *GC3 *and *Fpr *were statistically significantly greater for the highly expressed as opposed to the lowly expressed genes in both *Z. mays *and *T. aestivum *and for the male-specific specific as compared to flower-specific genes in *B. napus*. Notably, lowly-expressed female-specific genes have a statistically significant higher *GC3 *and *Fpr *than the highly expressed male-specific genes in *Z. mays *and no difference was detected between these two groups for *T. aestivum*. Most of the statistically significant comparisons (of 35 pairwise comparisons in total) remained significant after Bonferroni correction except for some contrasts among genes for high versus low expression within a gender (i.e., male-specific tissue/gametes in *Z. mays *and *T. aestivum *and within the flower in *B. napus*) and a single between gender comparison for *Fpr *at high expression levels in *T. aestivum *(*GC3 *for this comparison remained statistically significant). All other between gender comparisons remained statistically significant.

**Figure 2:**
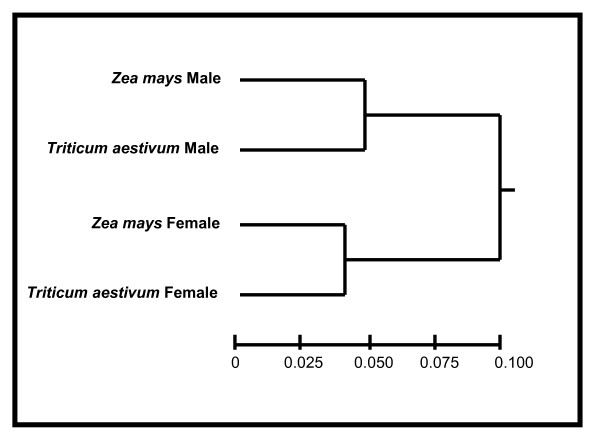
Hierarchical clustering based on Pearson correlation matrixes on relative synonymous codon usage values derived from concatenated EST sequences for each combination of species and gender (*Zea mays *and *Triticum aestivum *male-specific and female-specific genes).

The relative synonymous codon usage (*RSCU*) represents the observed frequency of a codon divided by the expected frequency (i.e., if all synonymous codons were used equally) [19]. Values different from 1, thus indicate the presence of bias. Analysis of *RSCU *for genes with male-specific and female-specific expression (concatenated across all genes) indicated that the bias in codon usage towards species-specific preferred codons was consistently higher in the female than in male tissues/gametes for *Z. mays *and *T. aestivum *(Table 2). In particular, 26 of 27 of the species-specific preferred codons for 18 amino acids in *Z. mays *(i.e, 18 have synonymous codons, Table 2) [42] were more frequent in female-specific than in male-specific genes. This represents a higher usage of at least one species-specific preferred codon for every amino acid with synonymous codons (noting that some amino acids have more than one preferred codon). For *T. aestivum*, female-specific genes had a greater usage of all 23 of the species-specific preferred codons (for 22 of 23 comparisons, the difference in *RSCU *was greater than 0.1). In addition, hierarchical clustering was conducted using Pearson correlation coefficients between *RSCU *values for each combination of species and gender-specific tissues/gametes for *Z. mays *and *T. aestivum *[43]. The results indicate that these groups cluster by gender rather than by species, consistent with gender being a major parameter in shaping codon usage (Figure 2). The *RSCU *data also show that *B. napus *demonstrates a preference towards GC ending codons in male-specific as compared to flower-specific genes (Table 2). The entire dataset across all species consisted of 1 497 876 codons.

**Table 2:** Relative synonymous codon usage (*RSCU*) as determined from concatenated EST sequences of female- and male-specific genes in *Zea mays *and *Triticum aestivum *and male-specific and flower-specific sequences in *Brassica napus*.

Amino acid	Codon	*RSCU*^a^										
		*Zea mays*				*Triticum aestivum*				*Brassica napus*		
				
		Female	Male	Difference (female minus male) ^b^	Codon(s) previously identified as Preferred^c^	Female	Male	Difference (female minus male)	Codon(s) previously identified as Preferred^c^	Male	Flower	Difference (male minus flower)^d^
Phe	UUU	0.65	0.86	-0.21		0.60	0.82	-0.22		0.83	0.90	-0.07
	UUC	1.35	1.14	**+0.21**	*	1.40	1.18	**+0.22**	*	1.17	1.10	+0.07
Leu	UUA	0.26	0.43	-0.17		0.27	0.42	-0.15		0.49	0.61	-0.12
	UUG	0.87	1.07	-0.20	*	0.79	0.98	-0.19		1.26	1.27	-0.01
	CUU	1.13	1.30	-0.17		0.98	1.28	-0.30		1.44	1.46	-0.02
	CUC	1.61	1.23	**+0.38**	*	1.82	1.39	**+0.43**	*	1.46	1.29	**+0.17**
	CUA	0.47	0.56	-0.09		0.39	0.49	-0.10		0.52	0.60	-0.08
	CUG	1.66	1.40	**+0.26**	*	1.77	1.44	**+0.33**		0.82	0.76	+0.06
Ile	AUU	0.92	1.11	-0.19		0.84	1.07	-0.23		1.06	1.05	+0.01
	AUC	1.57	1.22	**+0.35**	*	1.65	1.27	**+0.38**	*	1.39	1.26	**+0.13**
	AUA	0.51	0.67	-0.16		0.52	0.65	-0.13		0.55	0.69	-0.14
Val	GUU	0.96	1.25	-0.29		0.89	1.19	-0.30		1.43	1.46	-0.03
	GUC	1.26	1.07	**+0.19**	*	1.30	1.10	**+0.20**	*	0.95	0.89	+0.06
	GUA	0.35	0.46	-0.11		0.33	0.43	-0.10		0.39	0.47	-0.08
	GUG	1.44	1.22	**+0.22**	*	1.49	1.28	**+0.21**	*	1.22	1.17	+0.05
Ser	UCU	0.98	1.20	-0.22		0.88	1.18	-0.30		1.52	1.54	-0.02
	UCC	1.23	1.02	**+0.21**	*	1.37	1.13	**+0.24**	*	1	0.88	**+0.12**
	UCA	0.93	1.26	-0.33		0.88	1.13	-0.25		1	1.11	-0.11
	UCG	0.75	0.61	**+0.14**	*	0.90	0.63	**+0.27**		0.75	0.68	+0.07
	AGU	0.61	0.82	-0.21		0.57	0.77	-0.2		0.77	0.84	-0.07
	AGC	1.51	1.09	**+0.42**	*	1.40	1.16	**+0.24**	*	0.96	0.95	+0.01
Pro	CCU	1.07	1.27	-0.20		0.93	1.18	-0.25		1.55	1.49	+0.06
	CCC	0.99	0.73	**+0.26**	*	1.07	0.84	**+0.23**	*	0.63	0.57	+0.06
	CCA	1.04	1.35	-0.31		0.92	1.21	-0.29		1.08	1.19	-0.11
	CCG	0.90	0.65	**+0.25**	*	1.08	0.78	**+0.30**	*	0.73	0.76	-0.03
Thr	ACU	0.92	1.13	-0.21		0.80	1.06	-0.26		1.26	1.25	+0.01
	ACC	1.41	1.05	**+0.36**	*	1.51	1.17	**+0.34**	*	1.07	0.96	**+0.11**
	ACA	0.91	1.21	-0.30		0.83	1.15	-0.32		0.93	1.09	-0.16
	ACG	0.77	0.60	**+0.17**	*	0.86	0.62	**+0.24**	*	0.74	0.71	+0.03
Ala	GCU	1.13	1.34	-0.21		0.87	1.19	-0.32		1.74	1.69	+0.05
	GCC	1.23	0.99	**+0.24**	*	1.44	1.12	**+0.32**	*	0.76	0.72	+0.04
	GCA	0.88	1.07	-0.19		0.74	1	-0.26		0.81	0.96	-0.15
	GCG	0.77	0.60	**+0.17**	*	0.96	0.68	**+0.28**		0.68	0.64	+0.04
Tyr	UAU	0.65	0.88	-0.23		0.63	0.82	-0.19		0.78	0.83	-0.05
	UAC	1.35	1.12	**+0.23**	*	1.37	1.18	**+0.19**	*	1.22	1.17	+0.05
His	CAU	0.85	1.08	-0.23		0.77	0.99	-0.22		0.99	1.07	-0.08
	CAC	1.15	0.92	**+0.23**	*	1.23	1.01	**+0.22**	*	1.01	0.93	+0.08
Gln	CAA	0.63	0.77	-0.14		0.57	0.73	-0.16		0.89	0.98	-0.09
	CAG	1.37	1.23	**+0.14**	*	1.43	1.27	**+0.16**	*	1.11	1.02	+0.09
Asn	AAU	0.68	0.97	-0.29		0.71	0.92	-0.21		0.76	0.79	-0.03
	AAC	1.32	1.03	**+0.29**	*	1.29	1.08	**+0.21**	*	1.24	1.21	+0.03
Lys	AAA	0.50	0.70	-0.20		0.49	0.64	-0.15		0.72	0.88	-0.16
	AAG	1.50	1.30	**+0.20**	*	1.51	1.36	**+0.15**	*	1.28	1.12	**+0.16**
Asp	GAU	0.87	1.09	-0.22		0.82	1.04	-0.22		1.18	1.21	-0.03
	GAC	1.13	0.91	**+0.22**	*	1.18	0.96	**+0.22**	*	0.82	0.79	+0.03
Glu	GAA	0.63	0.83	-0.20		0.60	0.75	-0.15		0.81	0.90	-0.09
	GAG	1.37	1.17	**+0.20**	*	1.40	1.25	**+0.15**	*	1.19	1.10	+0.09
Cys	UGU	0.58	0.77	-0.19		0.51	0.73	-0.22		0.98	1.01	-0.03
	UGC	1.42	1.23	**+0.19**	*	1.49	1.27	**+0.22**	*	1.02	0.99	+0.03
Arg	CGU	0.66	0.77	-0.11		0.60	0.72	-0.12		1.08	0.98	**+0.10**
	CGC	1.29	1.06	**+0.23**	*	1.57	1.21	**+0.36**	*	0.68	0.54	**+0.14**
	CGA	0.41	0.55	-0.14		0.34	0.46	-0.12		0.54	0.58	-0.04
	CGG	0.89	0.77	**+0.12**		1.06	0.91	**+0.15**		0.45	0.51	-0.06
	AGA	0.90	1.17	-0.27		0.84	1.13	-0.29		1.71	1.92	-0.21
	AGG	1.84	1.68	**+0.16**	*	1.58	1.56	+0.02	*	1.54	1.48	+0.06
Gly	GGU	0.86	1.03	-0.17		0.74	0.97	-0.23		1.27	1.22	+0.05
	GGC	1.41	1.13	**+0.28**	*	1.65	1.30	**+0.35**	*	0.66	0.64	+0.02
	GGA	0.86	1.02	-0.16		0.71	0.92	-0.21		1.34	1.41	-0.07
	GGG	0.88	0.83	-0.05		0.90	0.82	+0.08		0.72	0.73	-0.01

Positive differences in *RSCU *greater than 0.1 in the tissue comparisons (highlighting codon preferences in female tissues) are in bold. Start and termination codons and the single codon for tryptophan have been excluded. Preferred codon data is not available for *B napus*.

^a ^The total number of codons used to estimate *RSCU*: *Zea mays *116 919 (sperm) and 94 794 (egg), *Triticum aestivum *453 286 (anther) and 199 397 (ovary) and *Brassica napus *232 516 (microspore) and 400 964 (flower). *RSCU *indicates the difference from a 1:1 ratio for all available codons within synonymous codon groups.

^b ^+, higher *RSCU *in female-specific genes; -, higher *RSCU *in male-specific genes.

^c ^Previously described as preferred codons in *Z. mays *and *T. aestivum *as indicated by their frequencies in high-versus low-biased genes by Kawabe and Miyashita (2003) [42]. Asterisk (*) indicates a preferred codon. No values available for *B. napus*.

^d ^+, higher *RSCU *in male-specific genes; -, higher *RSCU *in flower-specific genes.

Given that bias in codon usage has been inversely associated with protein length in certain eukaryotic organisms [e.g., [8]], we assessed whether it played a role in the observed gender-specific bias described above. For this, we identified the protein length for each gene under study (number of amino acids) in the *Arabidopsis thaliana *protein sequence database (i.e., genes previously identified as a match, see above). We used protein lengths from *Arabidopsis *because our DNA sequence data was derived from ESTs (that are partial sequences, not containing gene or protein length information) and because, unlike well-studied model organisms, the complete and/or annotated genomic DNA or protein sequences are not yet available for most of the genes in these plants. Protein lengths tend to be highly conserved among eukaryotes [44]. The data indicates that the mean protein length was greater for male-specific genes than female-specific genes in *Z. mays *and *T. aestivum *and for flower-specific genes as compared to male-specific genes in *B. napus *(mean protein length (± Standard Error): *Z. mays *egg 409.1 (± 13.2), *Z. mays *sperm 493.2 (± 13.3), *T. aestivum *ovary 426.3 (± 8.1), *T. aestivum *anther 571.3 (± 6.7), *B. napus *microspore 370 (± 6.7), *B. napus *flower 441.3 (± 5.6)). The higher mean lengths generally resulted from the presence of a relatively few genes that encoded very long proteins (between 1000 and 5000 amino acids). The number of genes encoding proteins with more than 1000 amino acids relative to the total number of genes are: *Zea mays *egg 38/946 = 4.0%,. *Z. mays *sperm 80/955 = 8.3%, *T. aestivum *ovary 69/1489 = 4.6%, *T. aestivum *anther 368/3326 = 11.1%, *B. napus *microspore 51/1675 = 3.0%, *B. napus *flower 176/3181 = 5.5%. We thus determined *RSCU *values for genes encoding proteins of similar lengths, in order to assess whether this protein length variation was related to our findings of gender-specific biases in codon usage. For this, male-specific and female-specific genes from each species under study (and *B. napus *flower-specific genes) were classified as being of either short (≤200 amino acids), medium (>200 and ≥400 amino acids) or of long length (>400 amino acids).

Comparisons were conducted for *RSCU *values relative to gender for *Z. mays *and *T. aestivum *(and male-versus flower-specific genes in *B. napus*) for the concatenated EST sequences (longest EST per contig) within each protein length category. The results show that female-specific genes consistently have higher values for *RSCU *for the preferred codons (as indicated by "+" sign in each of the columns) within each of these three protein length categories for *Z. mays *and *T. aestivum *(Table 3) [see Additional data file 1; Tables 1 to 3)] indicating that protein length variation does not explain the gender-specific bias observed for specific codons described in Table 2 (see Table 3). The bias in codon usage for male-specific versus flower-specific genes in *B. napus *was most evident for genes encoding long proteins.

**Table 3:** Difference in the relative synonymous codon usage (*RSCU*) for short genes (less than or equal to 200 amino acids in the homologous *Arabidopsis thaliana *protein), medium length genes (more than 200 amino acids and less than or equal to 400) and long genes (more 400 amino acids) as determined from concatenated EST sequences of female- and male-specific sequences in *Zea mays *and *Triticum aestivum *and male-specific and flower-specific sequences in *Brassica napus*. [Supplementary-material S1]

Amino acid	Codon	Difference in *RSCU *(Female-specific genes minus male-specific genes)^a^								Difference in *RSCU *(Male-specific genes minus flower-specific genes)^b^		
			
		*Zea mays*^c^				*Triticum aestivum*^c^				*Brassica napus*^c^		
				
		Short	Medium	Long	Codon(s) previously identified as preferred^d^	Short	Medium	Long	Codon(s) previously identified as preferred^d^	Short	Medium	Long
Phe	UUU	-0.15	-0.19	-0.23		-0.06	-0.12	-0.24		+0.05	-0.01	-0.21
	UUC	**+0.15**	**+0.19**	**+0.23**	*	**+0.06**	**+0.12**	**+0.24**	*	-0.05	+0.01	+0.21
Leu	UUA	-0.07	-0.15	-0.19		-0.06	-0.09	-0.14		-0.03	-0.01	-0.28
	UUG	-0.22	-0.20	-0.16	*	-0.01	-0.13	-0.15		+0.13	-0.05	-0.02
	CUU	-0.22	-0.10	-0.18		-0.17	-0.20	-0.30		+0.09	-0.09	-0.06
	CUC	**+0.50**	**+0.34**	**+0.27**	*	**+0.24**	**+0.24**	**+0.32**	*	**-0.18**	**-0.05**	**+0.59**
	CUA	-0.06	-0.10	-0.07		-0.03	-0.04	-0.10		-0.06	+0.02	-0.17
	CUG	**+0.07**	**+0.23**	**+0.32**	*	**+0.04**	**+0.23**	**+0.37**		+0.05	+0.18	-0.04
Ile	AUU	-0.14	-0.21	-0.18		-0.16	-0.14	-0.22		+0.08	+0.06	-0.08
	AUC	**+0.24**	**+0.33**	**+0.35**	*	**+0.12**	**+0.25**	**+0.33**	*	**-0.09**	**-0.07**	**+0.46**
	AUA	-0.09	-0.13	-0.17		+0.03	-0.12	-0.11		0	+0.01	-0.38
Val	GUU	-0.32	-0.22	-0.30		-0.20	-0.14	-0.30		+0.06	-0.07	-0.05
	GUC	**+0.25**	**+0.22**	**+0.08**	*	**+0.06**	**+0.13**	**+0.15**	*	-0.13	+0.01	+0.20
	GUA	-0.17	-0.08	-0.09		-0.04	-0.06	-0.10		-0.02	+0.03	-0.20
	GUG	**+0.23**	**+0.08**	**+0.31**	*	**+0.18**	**+0.07**	**+0.25**	*	+0.08	+0.03	+0.05
Ser	UCU	-0.19	-0.14	-0.28		-0.13	-0.17	-0.32		-0.01	-0.06	-0.01
	UCC	**+0.19**	**+0.11**	**+0.22**	*	**+0.19**	**+0.19**	**+0.13**	*	**-0.06**	**+0.02**	**+0.30**
	UCA	-0.33	-0.38	-0.25		-0.09	-0.28	-0.15		+0.04	+0.05	-0.34
	UCG	**+0.11**	**+0.10**	**+0.15**	*	**+0.13**	**+0.23**	**+0.25**		+0.02	-0.02	+0.15
	AGU	-0.19	-0.18	-0.20		-0.14	-0.13	-0.17		+0.09	-0.02	-0.21
	AGC	**+0.41**	**+0.47**	**+0.36**	*	**+0.04**	**+0.16**	**+0.25**	*	-0.09	+0.03	+0.10
Pro	CCU	-0.28	-0.19	-0.14		-0.10	-0.14	-0.24		+0.15	+0.03	+0.05
	CCC	**+0.29**	**+0.26**	**+0.18**	*	**+0.22**	**+0.19**	**+0.13**	*	-0.01	+0.05	+0.10
	CCA	-0.25	-0.23	-0.33		-0.22	-0.24	-0.21		-0.03	+0.03	-0.25
	CCG	**+0.23**	**+0.17**	**+0.28**	*	**+0.10**	**+0.19**	**+0.31**	*	-0.10	-0.12	+0.10
Thr	ACU	-0.21	-0.18	-0.21		-0.01	-0.26	-0.25		+0.09	+0.01	-0.01
	ACC	**+0.29**	**+0.33**	**+0.31**	*	**+0.13**	**+0.25**	**+0.28**	*	**-0.10**	**-0.06**	**+0.37**
	ACA	-0.17	-0.30	-0.30		-0.11	-0.19	-0.29		+0.01	+0.04	-0.43
	ACG	**+0.10**	**+0.14**	**+0.20**	*	**-0.01**	**+0.19**	**+0.27**	*	0	+0.01	+0.07
Ala	GCU	-0.11	-0.21	-0.22		-0.19	-0.27	-0.28		+0.18	0	+0.02
	GCC	**+0.18**	**+0.19**	**+0.25**	*	**+0.22**	**+0.27**	**+0.22**	*	-0.15	0	+0.16
	GCA	-0.10	-0.19	-0.19		-0.11	-0.18	-0.23		-0.04	+0.01	-0.33
	GCG	**+0.03**	**+0.19**	**+0.17**	*	**+0.08**	**+0.18**	**+0.29**		+0.02	-0.01	+0.15
Tyr	UAU	-0.13	-0.28	-0.19		-0.11	-0.08	-0.21		+0.06	+0.07	-0.24
	UAC	**+0.13**	**+0.28**	**+0.19**	*	**+0.11**	**+0.08**	**+0.21**	*	-0.06	-0.07	+0.24
His	CAU	-0.27	-0.22	-0.16		-0.03	-0.13	-0.20		+0.04	-0.01	-0.21
	CAC	**+0.27**	**+0.22**	**+0.16**	*	**+0.03**	**+0.13**	**+0.20**	*	-0.04	+0.01	+0.21
Gln	CAA	-0.11	-0.10	-0.15		-0.10	-0.10	-0.14		-0.09	-0.03	-0.17
	CAG	**+0.11**	**+0.10**	**+0.15**	*	**+0.10**	**+0.10**	**+0.14**	*	+0.09	+0.03	+0.17
Asn	AAU	-0.32	-0.32	-0.21		-0.01	-0.11	-0.19		+0.05	+0.06	-0.15
	AAC	**+0.32**	**+0.32**	**+0.21**	*	**+0.01**	**+0.11**	**+0.19**	*	-0.05	-0.06	+0.15
Lys	AAA	-0.14	-0.17	-0.20		-0.03	-0.10	-0.12		-0.09	-0.08	-0.27
	AAG	**+0.14**	**+0.17**	**+0.20**	*	**+0.03**	**+0.10**	**+0.12**	*	**+0.09**	**+0.08**	**+0.27**
Asp	GAU	-0.06	-0.21	-0.24		-0.09	-0.13	-0.20		+0.04	-0.01	-0.10
	GAC	**+0.06**	**+0.21**	**+0.24**	*	**+0.09**	**+0.13**	**+0.20**	*	-0.04	+0.01	+0.10
Glu	GAA	-0.08	-0.20	-0.20		-0.05	-0.07	-0.13		-0.08	-0.02	-0.16
	GAG	**+0.08**	**+0.20**	**+0.20**	*	**+0.05**	**+0.07**	**+0.13**	*	+0.08	+0.02	+0.16
Cys	UGU	-0.08	-0.14	-0.20		-0.10	-0.18	-0.17		+0.04	+0.02	-0.14
	UGC	**+0.08**	**+0.14**	**+0.20**	*	**+0.10**	**+0.18**	**+0.17**	*	-0.04	-0.02	+0.14
Arg	CGU	-0.01	-0.16	-0.07		-0.14	-0.12	-0.07		**+0.12**	**-0.05**	**+0.22**
	CGC	**+0.14**	**+0.17**	**+0.14**	*	**+0.06**	**+0.33**	**+0.20**	*	**+0.02**	**-0.01**	**+0.22**
	CGA	+0.01	-0.18	-0.12		-0.03	-0.08	-0.12		-0.11	+0.03	-0.03
	CGG	**+0.03**	**+0.08**	**+0.18**		**+0.18**	**+0.10**	**+0.18**		-0.05	-0.01	-0.10
	AGA	-0.21	-0.18	-0.26		-0.03	-0.15	-0.27		+0.04	+0.03	-0.57
	AGG	**+0.04**	**+0.28**	**+0.12**	*	-0.05	-0.07	+0.07	*	-0.02	-0.01	+0.20
Gly	GGU	-0.11	-0.11	-0.21		-0.16	-0.11	-0.23		+0.08	+0.05	+0.03
	GGC	**+0.34**	**+0.16**	**+0.31**	*	**+0.20**	**+0.29**	**+0.29**	*	-0.15	+0.01	+0.12
	GGA	-0.22	-0.08	-0.16		-0.07	-0.19	-0.18		-0.04	-0.06	-0.06
	GGG	0	+0.04	+0.07		+0.04	+0.01	+0.12		+0.11	0	-0.09

Start and termination codons and the single codon for tryptophan have been excluded. Preferred codon data is not available for B napus. Data for codons previously identified as having codon biases (bold in Table 2) are in bold. [See Additional data file 1 (Tables 1 to 3) for the full data].

^a ^+, higher *RSCU *in female-specific genes; -, higher *RSCU *in male-specific genes.

^b ^+, higher *RSCU *in male-specific genes; -, higher *RSCU *in flower-specific genes.

^c ^The total number of short genes and the average gene length (± Standard error), respectively, used to estimate *RSCU*: *Z. mays *sperm 149, 137.9 (± 3.2), *Z. mays *egg 204, 135.3 (± 2.9), *T. aestivum *anther 340, 142.3 (± 2.0), *T. aestivum *ovary 309, 138.8 (± 2.3), *B. napus *microspore 485, 144.8 (± 1.5), *B. napus *flower 598, 140.6 (± 1.6). The total number of medium length genes and the average gene length, respectively, used to estimate *RSCU*: *Z. mays *sperm 330, 295.2 (± 3.1), *Z. mays *egg 352, 296.2 (± 3.2), *T. aestivum *anther 893, 309.0 (± 1.9), *T. aestivum *ovary 520, 298.4 (± 2.6), *B. napus *microspore 608, 294.3 (± 2.4), *B. napus *flower 1127, 297.5 (± 1.7). The total number of long genes and the average gene length, respectively, used to estimate *RSCU*: *Z. mays *sperm 476, 741.6 (± 20.7), *Z. mays *egg 390, 655.4 (± 18.9), *T. aestivum *anther 2093, 753.4 (± 8.3), *T. aestivum *ovary 660, 661.7 (± 13.0), *B. napus *microspore 582, 639.5 (± 12.6), *B. napus *flower 1456, 676.1 (± 8.6).

^d ^Previously described as preferred codons in *Z. mays *and *T. aestivum *as indicated by their frequencies in high-versus low-biased genes by Kawabe and Miyashita (2003) [42]. Asterisk (*) indicates a preferred codon. No values available for *B. napus*.

Given that the codons showing bias generally ended in G or C for each of the species examined (Table 2), we also compared the *GC3 *content between each of the two tissue/gamete types per species for each of the protein length categories (Table 4). The results of pairwise comparisons show that the *GC3 *values are statistically significantly higher for female-specific than male-specific genes in *Z. mays *and *T. aestivum *and for male-specific than flower-specific genes in *B. napus *within each of the protein length categories, consistent with a gender-bias on codon usage. Examination of only those genes encoding very long proteins (equal to or more than 1000 amino acids) showed similar trends for each of these species. Notably, *GC3 *values were inversely correlated with protein length within each of the six species and tissue-specific datasets (i.e., for the male-specific, female-specific and for flower-specific datasets, Table 4) [see also Additional data file 1; Table 4], consistent with the relationship between codon usage and protein length reported in other species [8].

**Table 4:** Mean *GC3 *values for short genes (less than or equal to 200 amino acids in the homologous *Arabidopsis thaliana *protein), medium length genes (more than 200 amino acids and less than or equal to 400) and long genes (more 400 amino acids), and for genes encoding very long proteins only (equal to or more than 1000 amino acids) for female-specific and male-specific genes in *Zea mays *and *Triticum aestivum *and for male-specific and flower-specific genes in *Brassica napus*. [Supplementary-material S1]

	*Short*			*Medium*			*Long*			*Very long only*^a^		
				
	Female	Male	*P*-value	Female	Male	*P*-value	Female	Male	*P*-value	Female	Male	*P*-value
*Zea mays*	0.702	0.632	**<1.0 × 10**^-16^	0.635	0.542	**<1.0 × 10**^-16^	0.593	0.483	**<1.0 × 10**^-16^	0.505	0.656	***0.044***
*Triticum aestivum*	0.768	0.729	***0.015***	0.677	0.617	**<1.0 × 10**^-16^	0.612	0.526	**<1.0 × 10**^-16^	0.518	0.436	**<1.0 × 10**^-16^

*Brassica napus*	Male	Flower		Male	Flower		Male	Flower		Male	Flower	
	0.523	0.501	**0.002**	0.489	0.472	**0.0002**	0.469	0.448	**<1.0 × 10**^-16^	0.441	0.423	0.340

P-values are those for the Mann-Whitney Rank Sum Test (t-tests yielded similar P-values). P-values less than 0.05 are in bold. P-values not remaining statistically significant after Bonferroni correction among these contrasts are bold italics. For P-values of comparisons between protein length categories [see Additional data file 1 (Table 4)].

^a^, Sample sizes are relatively small for this group, influencing *P*-values for *Z. mays *and *B. napus*; the direction and magnitude of the differences are maintained for each comparison (see text). Mean protein lengths (± Standard Error) for this category are as follows. *Z. mays *sperm 1556.0 (± 63), *Z. mays *egg 1485.2 (± 112), *T. aestivum *anther 1377.0 (± 24.7), *T. aestivum *ovary 1334.7 (± 72.8), *B. napus *microspore 1370.5 (± 70.6), *B. napus *flower 1361.4 (± 34.0). Other mean protein length values are presented in Table 3.

**Figure 3:**
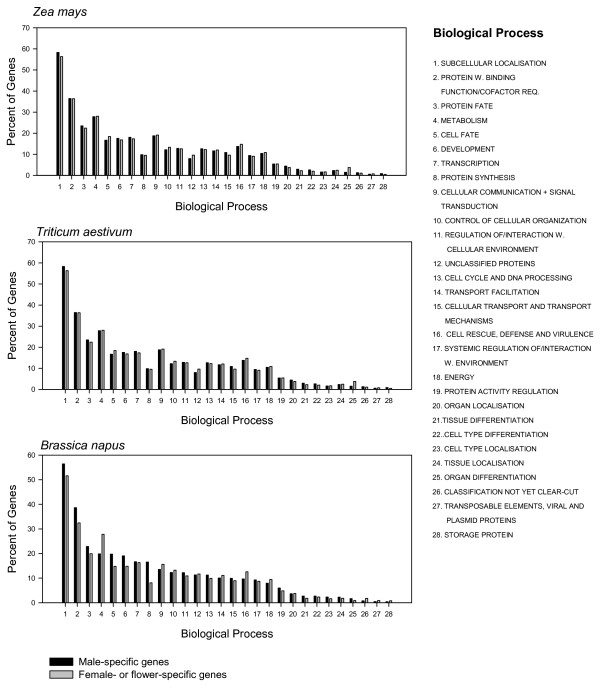
Biological functions of genes expressed only in the sperm and eggs in *Zea mays*, in the anther and ovary in *Triticum aestivum *and in the microspore and flower in *Brassica napus*. Sum of percentages may exceed 100 as some genes belong to more than one category.

Biological function has been proposed as a potential factor altering certain molecular evolutionary processes [10]; therefore we examined the gene profiles for each of the contrasting tissues for each species under study. The profile of biological functions of genes expressed only in the female tissues/gametes and male tissues/gametes were nearly identical for *Z. mays *and for *T. aestivum *(Figure 3). Similarly, the biological functions for genes specifically expressed in male microspores and those expressed in flowers were strongly associated in *B. napus*.

## Discussion

### Gender differences in codon usage

The statistically significant higher GC content at third codon positions and the greater frequency of preferred codons for genes expressed specifically in eggs as compared to sperm in *Z. mays *and for genes expressed in the ovary as compared to anther in *T. aestivum*, provide strong evidence that there is a greater bias in codon usage for genes expressed in female tissues than in male tissues and/or gametes (Figure 1). These findings were similar when genes were classified as either high expressing or low expressing, suggesting that gender has a substantial impact on synonymous codon use. We can infer that these differences are likely due to selective pressure because the bias is associated with gene expression (tissue-specific gene expression or high versus low expression level, Figure 1). Furthermore, the data indicate that the gender effect cannot be attributed to variation in protein lengths or to gene function (Table 3, Table 4, Figure 3). Overall, these results, across a broad range of genes, provide evidence that codon usage is altered by gender-specific pressures in plants.

Genes expressed in eggs have a higher relative synonymous codon usage value than those expressed in sperm for 26 of the 27 previously identified preferred codons for *Z. mays*, and indicates, remarkably, that selective pressure in eggs specifically acts to enhance the frequency of preferred codons for each of the 18 amino acids that have synonymous codons (i.e., egg-specific genes have a greater frequency of at least one of the preferred codons per amino acid, Table 2). Similar findings for *T. aestivum*, showing that all 23 of the preferred codons for this species are enhanced in genes expressed in the ovary as compared to the anther, also demonstrate that a selective pressure inherent to these female organs and gametes is acting to enhance the incidence of preferred codons across all synonymous codon groups. In addition, the fact that the female-bias was detected for each gene length category (i.e., among short genes, among medium length genes, and among long genes, Tables 3 and 4) and that the gender-specific gene expression was the major determinant of hierarchical clustering (relative to *RSCU *values), rather than species (Figure 2), supports the notion that the codon usage bias demonstrated here is greatly influenced by gender-specific factors. It is notable for *T. aestivum *that in three cases the *RSCU *was greater for female-than male-specific genes (bold values, no asterisk, Table 2) for G or C ending codons that had not been previously identified as preferred, but had been described as preferred in *Z. mays *and other plant species [42]. The overall results presented here, showing greater use of preferred codons in genes expressed in female organs and gametes, suggests that these codons are probably also preferred in *T. aestivum*, at least for genes expressed in the reproductive tissues and gametes.

The greater bias in codon usage among genes expressed in female organs and gametes as compared to male organs and gametes, reflecting an increased propensity for translational selection, could be caused by several factors. In particular, it is possible that protein products of genes expressed in female organs and gametes experience a more diverse biochemical environment than their male counterparts, a phenomenon that could lead to greater selective constraint on proteins [45,46], and thus, on their translation. It is also possible that mutations at third codon positions in genes expressed in female organs and gametes may on average have greater effects on fitness, as has been proposed for genes expressed across a broad array of tissues (higher selection coefficients) [8,9]. This could occur, for example, if translational inefficiency in female organs and gametes alters the cellular energy resources or interferes with essential biological processes in a manner not prevalent in male regions. Mutations affecting female regions could also have greater fitness effects because of the general uncertainty in the pollination process, which makes it highly advantageous for each ovary, ovule and/or egg to be fully functional (thereby mutations in female regions may affect fitness overall more than for anthers, pollen or sperm) [47] and because maternal traits can have a much greater impact on seed production (seed number, size, and dispersal) and survival (and thus, on overall fitness) [48]. Another possible explanation for the observed results is that there are differences in gene function between female and male organs and gametes, a theory that has been proposed as a potential factor altering amino acid substitution rates [10]. As shown in Figure 3, however, this is not the likely explanation in this study, as there is remarkable similarity in the biological functions represented by the male-specific and female-specific genes in both *Z. mays *and *T. aestivum*. Nonetheless, subtle differences in gene function (e.g., specific genes that influence codon usage) or other, unidentified, functional differences between the male and female tissues/gametes could play a role [10]. An additional potential contributing factor worth consideration is that genes that have greater breadth of expression throughout the entire plant (that can have greater bias in codon usage) [1], are coincidentally also more commonly expressed in female organs and gametes than in males. Although this possibility cannot be definitively excluded, it seems unlikely given the similarity between the functional profiles of female- and male-specific genes. Altogether, it seems that the best explanations are differences in the amount of selective pressure for effective translation due to different cellular environments and/or a greater impact of mutations on female tissues and gametes. Further studies will nonetheless be needed to ascertain the mechanisms underlying the greater bias in codon usage in female organs and gametes in these plant species.

The relationship between gender-specific gene expression and codon usage in *Z. mays *and *T. aestivum *is consistent with the very limited data currently available for other organisms. It has been shown in humans, for example, that genes expressed in ovaries have likely been under slightly greater selective constraint than testes for codon usage following the divergence of humans and mice [3]. The trend notably corresponds to the generally high rates of protein evolution (and thus reduced selective pressure) reported in genes involved in spermatogenesis in primates [49]. In *Drosophil*a, it has been found that the relative expression of genes in females versus males (the female : male ratio of gene expression) is well correlated to bias in codon usage [4]. In addition, in *Arabidopsis*, previous findings have indicated that more induced harmful mutations are passed to progeny by the sperm than by the eggs, consistent with the relatively lower selective pressure on mutations in male than in female tissues and gametes [7]. The present results extend these findings to include gender-specific selection on codon usage. Each of these gender-specific trends, in humans, *Drosophila*, and *Arabidopsis *are consistent with the findings we report here, and suggest that the higher bias in codon usage for genes expressed in female tissues could be inherent to a range of organisms. Further studies will be needed to better understand the full range of organisms for which gender-specific gene expression is associated with a bias in codon usage.

### Gene expression level

Gene expression level has been shown to be positively correlated with bias in codon usage in many organisms [1,8-15,24-27,31]. Selection is the best explanation for this finding because higher levels of gene expression lead to greater opportunity for selection to alter codon usage [8,10,12] and because mutational bias has only rarely been associated with gene expression level (in certain microorganisms) [8,50,51]. In *Drosophila*, a positive relationship between gene expression and bias in codon usage has been reported for female tissues, but a relatively weak negative correlation was detected for male tissues [4]. Our findings of greater values for *GC3 *and *Fpr *for highly expressed genes than for lowly expressed genes for both male-specific and female-specific genes from *Z. mays *and *T. aestivum *suggests that gene expression level is positively correlated to bias in codon usage for genes expressed in male and in female regions for these plants.

It is notable nonetheless that we found that the differences in the bias in codon usage between highly and lowly expressed genes were not as marked the male as in female tissues and gametes, as evidenced by the fact that the Bonferroni correction excluded the statistical significance of this comparison for both *Z. mays *and *T. aestivum*. In fact, the lowly expressed female-specific genes had statistically significantly higher bias in codon usage than the highly expressed male-specific genes in *Z. mays *and no difference was detected between these two groups for *T. aestivum *(Figure 1). It thus seems that female tissues/gametes maintain substantial selective pressure on codon usage even for genes with reduced expression, in a manner not characteristic of male tissues/gametes.

### Selection and gender-bias

The greater bias in codon usage for female-specific than for male-specific genes is currently best explained by selection. This is for the following reason. The male-specific and female-specific gene sets examined here were determined based on calculations that these genes were solely or primarily expressed in one tissue type and not in the other (i.e., the lack of ESTs in the contrasting tissue indicates that the mRNA was very rare or absent). Thus, the observed effects are associated with gender-specific gene expression (gene expression allows opportunity for selection, and is not usually associated with mutational bias [8,10,12]). In addition, our data indicate that gene function and protein length variation between male and female tissues/gametes do not explain the observed bias in codon usage between the gender-specific gene sets. Gene expression level differences cannot be implicated because the gender bias was detected for genes expressed at similar levels (high versus high expression and low versus low expression, regardless of protein length, Figure 1,) [see Additional data file 1, Table 5]. Notably, because we examined ESTs for the present analysis, which rarely contain introns, and studied plant species where annotated genomic DNA (containing the introns) is not yet available (NCBI, personal communication), we do not include an analysis of the GC (or AT) content of introns versus third codon positions in our genes, an approach sometimes used to exclude mutational bias [8,12,24,42,45,52]. Nevertheless, each of these trends, taken in their entirety, suggest that the bias in codon usage associated with gender-specific expression is best explained by differential selective pressure on genes expressed in male-specific tissues/gametes versus female-specific tissues/gametes.

### Male-specific versus flower-specific genes in *Brassica napus*

Although the differences in bias in codon usage between the microspore and flower in *B. napus *were generally lower in magnitude than the previous between gender comparisons for *Z. mays *and *T. aestivum *(Figure 1, Table 2), the data overall indicates that genes expressed in the two *B. napus *tissues have specific patterns of codon usage. Specifically, the higher GC content in *B. napus *microspore-specific genes than in flower-specific genes suggests that the male portion of the flower may be under more selective pressure for codon usage than the flower as a whole. In particular, given that the flower and flower bud EST library should represent genes from the male, female and vegetative (somatic) tissue, one can infer that the combined vegetative and female tissue is under less selective pressure than the microspore. Given that the vegetative tissue usually represents the greatest fraction of the flower tissue (petals, sepals) [53], then it could, in turn, be inferred that the somatic region is likely under reduced selective pressure for codon usage than the microspore (with no inference regarding the pressure in female tissues). The fact that the GC content at third codon positions of the genes specifically expressed in the microspores varied markedly among synonymous codon groups (Table 2), and was found to be positively correlated to gene expression level (Figure 1), further supports the notion that translational selection is enhanced in the microspore component of the flower. Moreover, from examination of Table 2, it is evident that for six of seven comparisons where the differences in *RSCU *between tissues were greater than 0.1, the microspore had enhanced usage of G or C ending codons (which were notably also the preferred codons in *Z. mays *and *T. aestivum*), a trend consistent with greater selective pressure. Notably, analysis of *RSCU *values relative to protein length suggests that differences between male-specific and flower-specific genes are greatest for genes encoding longer proteins (>400 amino acids, Table 3) in the *B. napus *tissue comparisons as these genes have substantially greater usage of G or C ending codons in this category ("+" signs in Table 3). This effect could be partially caused by the greater percentage of genes encoding very long proteins in the flower-specific dataset or by greater male-specific effects on codon usage for genes encoding longer proteins. Nonetheless, all three of the gene length categories demonstrate higher *GC3 *values for male-specific genes (Table 4). One possible interpretation of all of these findings in *B. napus*, when combined with the data from *Z. mays *and *T. aestivum*, is that the translational selection increases in the following order: flower-specific (heterogeneous) genes, male-specific genes, female-specific genes. Because these analyses are in different species, however, further evaluation of this possible relationship will be needed. Altogether, the totality of the findings here suggest that genes expressed in reproductive tissues may be under greater translational level selection than those expressed in vegetative (somatic) tissues, a factor consistent with the key role of reproductive success in fitness.

### Protein length and gene expression

The analysis of protein lengths indicates that genes encoding shorter proteins tend to generally have greater bias in codon usage, as indicated by *GC3*, for the species examined here (Table 4) [see also Additional data file 1; Table 4]. This is consistent with the trends reported in other organisms to date such as *Arabidopsis*, *Drosophila*, *C. elegans *and yeast [e.g., [8,54]]. We also found marked evidence that the gene expression level in the species studied here is inversely correlated to protein length for each of the six datasets examined (across all genes per dataset), a result consistent with trends reported in humans, *Drosophila *and *Populus tremula *[2,55,56]. In particular, the Pearson correlation coefficients were: *Z. mays *male (*R *= -0.135, *P *= 2.6 × 10^-5^), *Z. mays *female (*R *= -0.080, *P *= 0.010), *T*. *aestivum *male (*R *= -0.049, 4.3 × 10^-3^), *T. aestivum *female (*R *= -0.18, *P *= 1.1 × 10^-12^), *B. napus *male (*R *= -0.149, *P *= 1.9 × 10^-9^), and *B. napus *flower (*R *= -0.081, *P *= 4.6 × 10^-6^). This suggests that the tendency of shorter genes to have greater bias in codon usage (Table 4), at least for the genes examined here, may be due to greater levels of gene expression and an associated selective pressure [54]. Notably, in a complementary analysis to Figure 1, we found that the gender-bias in codon usage was evident among highly and among lowly expressed genes within each the three different protein length categories (short, medium, and long) [see Additional data file 1; Table 5].

Thus, the gender-specific biases in *Z. mays *and *T. aestivum *(and flower-specific differences in *B. napus*) at high and low levels of gene expression observed in Figure 1 cannot be explained by differences in protein lengths. In addition to the inverse association between protein length and bias in codon usage, it is also evident from Table 4 that the three species examined here tend to have different values for *GC3*, with decreasingly lower values occurring from *T. aestivum*, to *Z. mays *and to *B. napus*. Altogether, it is evident from our entire analysis that the gender-specific effects on codon usage can be detected across a range of protein lengths, gene expression levels, and for different plant species, thereby demonstrating that gender-specific factors play a significant role in genome evolution.

### Notable issues

It should be noted that the *B. napus *material used for the microspore cDNA library was grown at low temperatures (10°C/5°C) that could potentially alter some fraction of the gene expression in the microspore if stress-mediation genes were enhanced. In fact, we found less than 1% of the total genes in *B. napus *microspores were stress-related (data not shown). Another issue worth considering is the implication of previous findings of gender-specific mutation rates in plants, a trend that was based on the detection of higher evolutionary rates at silent sites, including third codon positions, in male gametes [57]. Higher mutation rates in sperm, however, should act to enhance per generational mutation rates across the entire genome, including those genes expressed in females and in males, and thus, not impact the observed bias in codon usage.

Nonetheless, it should be noted that the differential male/female inheritance of organelles (and the underlying mechanisms; pre- or post-zygotic), could influence whether these genes are expressed in male or female tissues/gametes, and potentially contribute to the codon usage for organellar genes and their substitution rates [57,58]). It is also notable that the abundance of tRNA of the preferred codons could be greater for female than the male tissues/gametes and contribute to the gender differences in codon usage [3,4]. This seems relatively unlikely given that the abundance of tRNAs would have to be higher in female-tissues/gametes for every single preferred codon and that such differences have been shown not to explain the gender-specific codon usage in *Drosophila *[4]. It should also be noted that the reproducibility of the results for *GC3 *and *Fpr *observed in this study is consistent with the notion that *GC3 *content alone could be an effective indicator of codon bias in some species [31].

## Conclusion

This study reports findings of female-specific bias in codon usage in plants. The remarkable consistency of the increased GC content of third codon positions and the increased frequency of preferred codons for both *Z. mays *and *T. aestivum*, even across different gene expression levels and protein lengths, combined with the enhanced usage of species-specific preferred codons for each of the 18 amino acids having synonymous codons, strongly indicate that gender plays a key role in codon usage. The findings in *B. napus *suggest that the tissues of the reproductive system, including both male and female organs, have a greater impact on codon usage than somatic regions. Overall, it is apparent that gender needs to be a key player in furthering our understanding of translational level selection. Further study will be needed to ascertain whether this is a generalized phenomenon, inherent to other organisms, as it could play a key role in DNA and protein sequence changes relevant to epidemiology, population genetics and to molecular evolution.

## Methods

### Sequence data

Sperm and egg EST libraries from *Z. mays *and anther and ovary libraries for *T. aestivum *were extracted from Genbank using Entrez Nucleotide available at the National Center for Biotechnology Information [41] (Table 1). We chose these data because of the availability of large gender-specific EST libraries (>4000) in Genbank. When more than one library was available we chose the one most likely to reflect gamete expression (e.g., an ovary library was selected over a pistil library in *T. aestivum*). In addition to these libraries, we also collected *B. napus *sequences from an in-house cDNA library representing isolated late-uninucleate and early-binucleate microspores (male germline cells) and from a publicly available flower library (Table 1). The EST datasets used represent those that were available to us as of March 2006.

### Expression profiles and preparation of sequence data

Each of the EST sequences from each of the six libraries was compared against the *A. thaliana *protein sequence database using BLASTX [39-41]. Only EST sequences having an e-value of less than 1 × 10^-7 ^to known or hypothetical proteins of *A. thaliana *were kept for further analysis. Notably, this process automatically excludes all rRNAs from the analysis as they would not be in the protein database. Using these datasets, we clustered and assembled the ESTs for each library into contigs and singletons using the software program CAP3 [38] (Table 1). The expression profile for each gene was determined from the number of ESTs per contig, and this value was 1 for singleton ESTs. For each gene in each of the six datasets, we standardized the expression level by dividing these values by the total number of ESTs in the original and complete EST library (Table 1), an approach that has been previously demonstrated to be an effective measure of expression level [8,12,31]. These values were multiplied by 10 000 (to obtain ESTs per 10 000) and the expression level for each gene was categorized as either high (>5 ESTs per 10 000) or low (≤5 ESTs per 10 000). Although many of the putative unigenes used here have not yet been definitively described as genes for those species, we nonetheless refer to them as "genes" here and in the text.

We identified genes that were expressed only in the male library and only in the female library (or flower for *B. napus*) as follows. Beginning with the male EST library from each species, the longest EST sequence per contig was identified and chosen as the representative for that gene. Each singleton represents its own gene. Each of these ESTs was then submitted to MEGABLAST [41] as a query against the original and redundant female-specific EST dataset (or flower library for *B. napus*, Table 1). The original female EST dataset was used in order to be conservative in the identification of male-specific ESTs. ESTs having more than 95% similarity were considered a match, which represents a level of similarity rigorous enough to distinguish among genes in conserved gene families [32]. The genes not having matches were categorized as male-specific. The process was then repeated for the female set of genes (or flower-specific for *B. napus*), after removing the sequences that were identified as matches to the male EST library. Specifically, the longest EST per contig and each singleton was queried against the original and redundant male-specific EST library in MEGABLAST. Genes that did not have matches were considered female-specific. The final datasets were: *Z. mays *sperm-specific genes (N = 955), *Z. mays *egg-specific genes (*N *= 946), *T. aestivum *anther-specific genes (*N *= 3326), *T. aestivum *ovary-specific genes (*N *= 1489), *B. napus *microspore-specific genes (*N *= 1675) and *B. napus *flower-specific genes (*N *= 1637). Genes with high expression (>5 ESTs per 10 000) represented approximately one quarter (or less) of each of these datasets. Notably, these gender-specific genes represent those that are specific to a particular tissue or gamete (e.g. sperm) when compared to only one other tissue or gamete (e.g., egg), and thus the tissue/gamete-specific genes identified here for each species are more numerous than one would observe if these had been compared to all the available libraries for that species (as is the standard analysis for ESTs). As well, the description of a gene as tissue-specific (e.g., female-specific) does not necessarily indicate that the gene is not expressed in the opposing tissue (e.g., male-specific) but rather that there were no ESTs present in the publicly available EST dataset used for comparison. It should be noted that although the genomic DNA sequences were available for a small portion of the ESTs for each species, we only used EST sequences for this entire analysis for consistency.

The open reading frame for each singleton and an EST representing every contig (longest EST per contig) was identified for every gene from each of the six reduced-sized gender-specific sequence datasets (see above) using alignments from BLASTX against the *A. thaliana *protein database. The BLASTX amino acid based algorithm provides alignments of the six-frame translated EST relative to the protein database and thereby is more sensitive to elements of functionality and homology than DNA alignments and accurately reveals reading frames [41,59]. Using the amino acid alignments between the translated ESTs and the *A. thalian*a homologues, we identified and extracted that portion of each EST sequence representing the reading frame (these generally did not include the start or termination codons). Most edited sequences were between 200 and 700 bp in length. Gaps in the alignments were rare, but when identified these regions were excluded from the EST, as were any occasional missense codons (resulting from the less than 1% sequencing errors in most large scale EST sequencing projects; in-house ESTs had PHRED scores [33] of greater than 20, representing less than a 1% error). All DNA sequence editing was conducted using BioEdit 7.0.5.3 [60].

### Data analysis

The GC content at third codon positions (*GC3*) and the frequency of preferred codons (*Fpr*) for each gene was determined using CodonW [61]. *GC3 *content has been shown to be well-correlated with the degree of biased codon usage for *A. thaliana *and other plant species [1,31]. For the determination of *Fpr*, we used the preferred codons (sometimes called favoured codons) for *Z. mays *and *T. aestivum *previously identified by Kawabe and Miyashita (2003) [41]. Preferred codons are those that are most frequently used in the most highly-biased genes (as compared to lowly-biased genes) per degenerate codon group, and have been well-correlated to the optimal codons for many species (those codons used in the most highly expressed genes) [8]. *Fpr *was not determined for *B. napus *as the preferred codon data have not been described yet (and cannot be determined here as then the same data would then have to be used to determine both the preferred codons and *Fpr*).

A series of pairwise comparisons were conducted with respect to each combination of gender and gene expression level using *GC3 *and *Fpr *(including the flower for *B. napus*). Additional pairwise contrasts were conducted to assess the impact of gene expression within each gender-specific dataset for each species (and the flower in *B. napus*) for a total of 35 contrasts. Tests were conducted using the non-parametric Mann-Whitney Rank Sum test (as normality was not detected in some contrasts; *t*-tests nonetheless yielded similar results). Statistical significance required *P *< 0.05. A Bonferroni correction was applied across all contrasts. All statistical analyses were conducted in SigmaPlot 10.0 and SigmaStat 3.5 for Windows (Systat^© ^software 2006). In addition to these pairwise tests, we also determined the relative synonymous codon usage (*RSCU*) for the concatenated reading frames for the entire dataset of male-specific and for female-specific (and the flower for *B. napus*) genes for each species using CodonW [61]. See main text for the description of gender-specific *RSCU*, *GC3*, and gene expression relative to protein length. Hierarchical clustering was conducted based on the Pearson correlation coefficients between *RSCU *values for each combination of species and gender-specific tissues/gametes for *Z. mays *and *T. aestivum *(Systat, 2004)[43].

As a means to assess whether the profile of gene functions conducted by male-specific and female-specific genes differed for *Z. mays *and for *T. aestivum*, each of these gender-specific sequence datasets (open reading frames) were submitted to MIPs [62]. A similar analysis was conducted for male-specific and flower-specific genes in *B. napus*. The gene functions were determined by comparison to proteins characterized in the *A. thaliana *database (MATDB)[62] with annotation from The Arabidopsis Information Resource (TAIR) and as implemented by the software Classification Superviewer [63].

## Authors' contributions

CAW conceived the project, conducted the majority of the bioinformatics and comparative analysis, and prepared the manuscript. MRM conducted laboratory work for the *B. napus *microspore EST library and participated in the analysis and the editing of the manuscript. JEK is the research group supervisor and contributed to the conception of the study, managed the data from the *B. napus *microspore library, and was involved in the data analysis and the editing of the manuscript. All authors read and approved the final manuscript.

## Supplementary Material

Additional File 1 That includes five Tables that support the data described in the main text. Click here for file
