# Supplementary material for: Gender-specific selection on codon usage in plant genomes
Source: BMC Genomics. 2007 Jun 13;8:169. doi: 10.1186/1471-2164-8-169 (PMC1919372; doi:10.1186/1471-2164-8-169)
Supplement: Additional File 1 — That includes five Tables that support the data described in the main text. [file 1471-2164-8-169-S1.pdf]

Table 1. Relative synonymous codon usage (*RSCU*) for short genes (less than 200 amino acids in the homologous *Arabidopsis thaliana* protein) as determined from concatenated EST sequences of female- and male-specific genes in *Zea mays* and *Triticum aestivum* and flower- and male-specific sequences in *Brassica napus*. Start and termination codons and the single codon for tryptophan have been excluded. Preferred codon data is not available for *B napus*.

Amino acid	Codon	*RSCU*[a]										
		Zea mays				*Triticum aestivum*				*Brassica napus*		
		Female	Male	Difference (female minus male)	Codon(s) previously identified as preferred[b]	Female	Male	Difference (female minus male)	Codon(s) previously identified as preferred[b]	Male	Flower	Difference (male minus flower)
Phe	UUU	0.50	0.65	-0.15		0.45	0.51	-0.06		0.84	0.79	+0.05
	UUC	1.50	1.35	+0.15	*	1.55	1.49	+0.06	*	1.16	1.21	-0.05
Leu	UUA	0.17	0.24	-0.07		0.13	0.19	-0.06		0.49	0.52	-0.03
	UUG	0.63	0.85	-0.22	*	0.53	0.54	-0.01		1.28	1.15	+0.13
	CUU	0.92	1.14	-0.22		0.76	0.93	-0.17		1.48	1.39	+0.09
	CUC	2.21	1.71	+0.50	*	2.52	2.28	+0.24	*	1.44	1.62	-0.18
	CUA	0.34	0.40	-0.06		0.23	0.26	-0.03		0.51	0.57	-0.06
	CUG	1.72	1.65	+0.07	*	1.84	1.80	+0.04		0.80	0.75	+0.05
Ile	AUU	0.80	0.94	-0.14		0.61	0.77	-0.16		1.09	1.01	+0.08
	AUC	1.84	1.60	+0.24	*	2.03	1.91	+0.12	*	1.35	1.44	-0.09
	AUA	0.37	0.46	-0.09		0.35	0.32	+0.03		0.56	0.56	0
Val	GUU	0.69	1.01	-0.32		0.62	0.82	-0.20		1.46	1.40	+0.06
	GUC	1.54	1.29	+0.25	*	1.55	1.49	+0.06	*	0.95	1.08	-0.13
	GUA	0.22	0.39	-0.17		0.21	0.25	-0.04		0.36	0.38	-0.02
	GUG	1.54	1.31	+0.23	*	1.62	1.44	+0.18	*	1.22	1.14	+0.08
Ser	UCU	0.84	1.03	-0.19		0.67	0.80	-0.13		1.54	1.55	-0.01
	UCC	1.53	1.34	+0.19	*	1.85	1.66	+0.19	*	1.00	1.06	-0.06
	UCA	0.67	1.00	-0.33		0.52	0.61	-0.09		0.98	0.94	+0.04
	UCG	0.88	0.77	+0.11	*	1.00	0.87	+0.13		0.77	0.75	+0.02

	AGU	0.40	0.59	-0.19		0.32	0.46	-0.14		0.79	0.70	+0.09
	AGC	1.68	1.27	+0.41	*	1.64	1.60	+0.04	*	0.91	1.00	-0.09
Pro	CCU	0.90	1.18	-0.28		0.68	0.78	-0.10		1.56	1.41	+0.15
	CCC	1.22	0.93	+0.29	*	1.43	1.21	+0.22	*	0.65	0.66	-0.01
	CCA	0.88	1.13	-0.25		0.67	0.89	-0.22		1.06	1.09	-0.03
	CCG	1.00	0.77	+0.23	*	1.22	1.12	+0.10	*	0.73	0.83	-0.10
Thr	ACU	0.71	0.92	-0.21		0.73	0.74	-0.01		1.24	1.15	+0.09
	ACC	1.77	1.48	+0.29	*	1.90	1.77	+0.13	*	1.09	1.19	-0.10
	ACA	0.74	0.91	-0.17		0.55	0.66	-0.11		0.92	0.91	+0.01
	ACG	0.79	0.69	+0.10	*	0.82	0.83	-0.01	*	0.75	0.75	0
Ala	GCU	0.97	1.08	-0.11		0.67	0.86	-0.19		1.78	1.60	+0.18
	GCC	1.42	1.24	+0.18	*	1.75	1.53	+0.22	*	0.77	0.92	-0.15
	GCA	0.73	0.83	-0.10		0.48	0.59	-0.11		0.79	0.83	-0.04
	GCG	0.88	0.85	+0.03	*	1.10	1.02	+0.08		0.66	0.64	+0.02
Tyr	UAU	0.49	0.62	-0.13		0.45	0.56	-0.11		0.78	0.72	+0.06
	UAC	1.51	1.38	+0.13	*	1.55	1.44	+0.11	*	1.22	1.28	-0.06
His	CAU	0.61	0.88	-0.27		0.55	0.58	-0.03		1.00	0.96	+0.04
	CAC	1.39	1.12	+0.27	*	1.45	1.42	+0.03	*	1.00	1.04	-0.04
Gln	CAA	0.51	0.62	-0.11		0.38	0.48	-0.10		0.88	0.97	-0.09
	CAG	1.49	1.38	+0.11	*	1.62	1.52	+0.10	*	1.12	1.03	+0.09
Asn	AAU	0.46	0.78	-0.32		0.51	0.52	-0.01		0.76	0.71	+0.05
	AAC	1.54	1.22	+0.32	*	1.49	1.48	+0.01	*	1.24	1.29	-0.05
Lys	AAA	0.33	0.47	-0.14		0.28	0.31	-0.03		0.72	0.81	-0.09
	AAG	1.67	1.53	+0.14	*	1.72	1.69	+0.03	*	1.28	1.19	+0.09
Asp	GAU	0.77	0.83	-0.06		0.60	0.69	-0.09		1.19	1.15	+0.04
	GAC	1.23	1.17	+0.06	*	1.40	1.31	+0.09	*	0.81	0.85	-0.04
Glu	GAA	0.51	0.59	-0.08		0.41	0.46	-0.05		0.80	0.88	-0.08
	GAG	1.49	1.41	+0.08	*	1.59	1.54	+0.05	*	1.20	1.12	+0.08
Cys	UGU	0.43	0.51	-0.08		0.29	0.39	-0.10		0.99	0.95	+0.04
	UGC	1.57	1.49	+0.08	*	1.71	1.61	+0.10	*	1.01	1.05	-0.04
Arg	CGU	0.60	0.61	-0.01		0.52	0.66	-0.14		1.13	1.01	+0.12
	CGC	1.81	1.67	+0.14	*	2.06	2.00	+0.06	*	0.70	0.68	+0.02
	CGA	0.41	0.40	+0.01		0.21	0.24	-0.03		0.50	0.61	-0.11
	CGG	0.85	0.82	+0.03		1.06	0.88	+0.18		0.43	0.48	-0.05

	AGA	0.58	0.79	-0.21		0.50	0.53	-0.03		1.71	1.67	+0.04
	AGG	1.75	1.71	+0.04	*	1.65	1.70	-0.05	*	1.53	1.55	-0.02
Gly	GGU	0.76	0.87	-0.11		0.58	0.74	-0.16		1.28	1.20	+0.08
	GGC	1.68	1.34	+0.34	*	1.89	1.69	+0.20	*	0.66	0.81	-0.15
	GGA	0.70	0.92	-0.22		0.60	0.67	-0.07		1.33	1.37	-0.04
	GGG	0.86	0.86	0		0.93	0.89	+0.04		0.73	0.62	+0.11

[a] The total number of short genes and the average gene length (±Standard error), respectively, used to estimate *RSCU*: *Z. mays* sperm 149, 137.9 (±3.2), *Z. mays* egg 204, 135.3 (±2.9), *T. aestivum* anther 340, 142.3 (±2.0), *T. aestivum* ovary 309, 138.8 (±2.3), *B. napus* microspore 485, 144.8 (±1.5), *B. napus* flower 598, 140.6 (±1.6).

[b] Previously described as preferred codons in *Z. mays* and *T. aestivum* as indicated by their frequencies in high- versus low-biased genes by Kawabe and Miyashita [42]. Asterisk (*) indicates a preferred codon. No values available for *B. napus*.

Table 2. Relative synonymous codon usage (*RSCU*) for medium length genes (between 200 and 400 amino acids in the homologous *Arabidopsis thaliana* protein) as determined from concatenated EST sequences of female- and male-specific genes in *Zea mays* and *Triticum aestivum* and flower- and male-specific sequences in *Brassica napus*. Start and termination codons and the single codon for tryptophan have been excluded. Preferred codon data is not available for *B napus*.

Amino acid	Codon	*RSCU*[a]										
		Zea mays				*Triticum aestivum*				*Brassica napus*		
		Female	Male	Difference (female minus male)	Codon(s) previously identified as preferred[b]	Female	Male	Difference (female minus male)	Codon(s) Previously Identified as Preferred[b]	Male	Flower	Difference (Male minus flower)
Phe	UUU	0.62	0.81	-0.19		0.56	0.68	-0.12		0.88	0.89	-0.01
	UUC	1.38	1.19	+0.19	*	1.44	1.32	+0.12	*	1.12	1.11	+0.01
Leu	UUA	0.25	0.40	-0.15		0.24	0.33	-0.09		0.57	0.58	-0.01
	UUG	0.81	1.01	-0.20	*	0.74	0.87	-0.13		1.25	1.30	-0.05
	CUU	1.13	1.23	-0.10		0.96	1.16	-0.20		1.40	1.49	-0.09
	CUC	1.68	1.34	+0.34	*	1.92	1.68	+0.24	*	1.31	1.36	-0.05
	CUA	0.49	0.59	-0.10		0.38	0.42	-0.04		0.57	0.55	+0.02
	CUG	1.65	1.42	+0.23	*	1.77	1.54	+0.23		0.89	0.71	+0.18
Ile	AUU	0.88	1.09	-0.21		0.80	0.94	-0.14		1.08	1.02	+0.06
	AUC	1.62	1.29	+0.33	*	1.74	1.49	+0.25	*	1.26	1.33	-0.07
	AUA	0.49	0.62	-0.13		0.46	0.58	-0.12		0.66	0.65	+0.01
Val	GUU	1.01	1.23	-0.22		0.87	1.01	-0.14		1.41	1.48	-0.07
	GUC	1.29	1.07	+0.22	*	1.38	1.25	+0.13	*	0.90	0.89	+0.01
	GUA	0.32	0.40	-0.08		0.32	0.38	-0.06		0.47	0.44	+0.03
	GUG	1.38	1.30	+0.08	*	1.43	1.36	+0.07	*	1.22	1.19	+0.03
Ser	UCU	1.01	1.15	-0.14		0.84	1.01	-0.17		1.50	1.56	-0.06
	UCC	1.29	1.18	+0.11	*	1.52	1.33	+0.19	*	0.95	0.93	+0.02
	UCA	0.86	1.24	-0.38		0.77	1.05	-0.28		1.10	1.05	+0.05
	UCG	0.74	0.64	+0.10	*	0.93	0.70	+0.23		0.68	0.70	-0.02

	AGU	0.57	0.75	-0.18		0.52	0.65	-0.13		0.81	0.83	-0.02
	AGC	1.52	1.05	+0.47	*	1.42	1.26	+0.16	*	0.96	0.93	+0.03
Pro	CCU	1.02	1.21	-0.19		0.90	1.04	-0.14		1.54	1.51	+0.03
	CCC	1.06	0.80	+0.26	*	1.16	0.97	+0.19	*	0.62	0.57	+0.05
	CCA	1.03	1.26	-0.23		0.83	1.07	-0.24		1.16	1.13	+0.03
	CCG	0.89	0.72	+0.17	*	1.11	0.92	+0.19	*	0.68	0.80	-0.12
Thr	ACU	0.95	1.13	-0.18		0.73	0.99	-0.26		1.28	1.27	+0.01
	ACC	1.43	1.10	+0.33	*	1.62	1.37	+0.25	*	0.92	0.98	-0.06
	ACA	0.83	1.13	-0.30		0.75	0.94	-0.19		1.08	1.04	+0.04
	ACG	0.79	0.65	+0.14	*	0.90	0.71	+0.19	*	0.73	0.72	+0.01
Ala	GCU	1.12	1.33	-0.21		0.78	1.05	-0.27		1.71	1.71	0
	GCC	1.23	1.04	+0.19	*	1.55	1.28	+0.27	*	0.72	0.72	0
	GCA	0.85	1.04	-0.19		0.70	0.88	-0.18		0.91	0.90	+0.01
	GCG	0.79	0.60	+0.19	*	0.97	0.79	+0.18		0.66	0.67	-0.01
Tyr	UAU	0.62	0.90	-0.28		0.62	0.70	-0.08		0.87	0.80	+0.07
	UAC	1.38	1.10	+0.28	*	1.38	1.30	+0.08	*	1.13	1.20	-0.07
His	CAU	0.83	1.05	-0.22		0.73	0.86	-0.13		1.04	1.05	-0.01
	CAC	1.17	0.95	+0.22	*	1.27	1.14	+0.13	*	0.96	0.95	+0.01
Gln	CAA	0.62	0.72	-0.10		0.54	0.64	-0.10		0.94	0.97	-0.03
	CAG	1.38	1.28	+0.10	*	1.46	1.36	+0.10	*	1.06	1.03	+0.03
Asn	AAU	0.63	0.95	-0.32		0.65	0.76	-0.11		0.82	0.76	+0.06
	AAC	1.37	1.05	+0.32	*	1.35	1.24	+0.11	*	1.18	1.24	-0.06
Lys	AAA	0.50	0.67	-0.17		0.45	0.55	-0.10		0.79	0.87	-0.08
	AAG	1.50	1.33	+0.17	*	1.55	1.45	+0.10	*	1.21	1.13	+0.08
Asp	GAU	0.85	1.06	-0.21		0.78	0.91	-0.13		1.19	1.20	-0.01
	GAC	1.15	0.94	+0.21	*	1.22	1.09	+0.13	*	0.81	0.80	+0.01
Glu	GAA	0.61	0.81	-0.20		0.57	0.64	-0.07		0.86	0.88	-0.02
	GAG	1.39	1.19	+0.20	*	1.43	1.36	+0.07	*	1.14	1.12	+0.02
Cys	UGU	0.59	0.73	-0.14		0.46	0.64	-0.18		1.02	1.00	+0.02
	UGC	1.41	1.27	+0.14	*	1.54	1.36	+0.18	*	0.98	1.00	-0.02
Arg	CGU	0.64	0.80	-0.16		0.55	0.67	-0.12		0.97	1.02	-0.05
	CGC	1.32	1.15	+0.17	*	1.76	1.43	+0.33	*	0.57	0.55	-0.01
	CGA	0.34	0.52	-0.18		0.34	0.42	-0.08		0.57	0.56	+0.03
	CGG	0.87	0.79	+0.08		1.05	0.95	+0.10		0.49	0.50	-0.01

	AGA	0.96	1.14	-0.18		0.80	0.95	-0.15		1.93	1.90	+0.03
	AGG	1.88	1.60	+0.28	*	1.51	1.58	-0.07	*	1.47	1.48	-0.01
Gly	GGU	0.83	0.94	-0.11		0.71	0.82	-0.11		1.26	1.21	+0.05
	GGC	1.42	1.26	+0.16	*	1.77	1.48	+0.29	*	0.65	0.64	+0.01
	GGA	0.87	0.95	-0.08		0.65	0.84	-0.19		1.35	1.41	-0.06
	GGG	0.88	0.84	+0.04		0.87	0.86	+0.01		0.74	0.74	0

[a] The total number of medium length genes and the average gene length, respectively, used to estimate *RSCU*: *Z. mays* sperm 330, 295.2 (±3.1), *Z. mays* egg 352, 296.2 (±3.2), *T. aestivum* anther 893, 309.0 (±1.9), *T. aestivum* ovary 520, 298.4 (±2.6), *B. napus* microspore 608, 294.3 (±2.4), *B. napus* flower 1127, 297.5 (±1.7).

[b] Previously described as preferred codons in *Z. mays* and *T. aestivum* as indicated by their frequencies in high- versus low-biased genes by Kawabe and Miyashita [42]. Asterisk (*) indicates a preferred codon. No values available for *B. napus*.

Table 3. Relative synonymous codon usage (*RSCU*) for long genes (more than 400 amino acids in the homologous *Arabidopsis thaliana* protein) as determined from concatenated EST sequences of female- and male-specific genes in *Zea mays* and *Triticum aestivum* and flower- and male-specific sequences in *Brassica napus*. Start and termination codons and the single codon for tryptophan have been excluded. Preferred codon data is not available for *B napus*.

Amino acid	Codon	*RSCU*[a]										
		Zea mays				*Triticum aestivum*				*Brassica napus*		
		Female	Male	Difference (female minus male)	Codon(s) Previously identified as preferred[b]	Female	Male	Difference (female minus male)	Codon(s) previously identified as preferred[b]	Male	Flower	Difference (male minus flower)
Phe	UUU	0.73	0.96	-0.23		0.68	0.92	-0.24		0.74	0.95	-0.21
	UUC	1.27	1.04	+0.23	*	1.32	1.08	+0.24	*	1.26	1.05	+0.21
Leu	UUA	0.31	0.50	-0.19		0.33	0.47	-0.14		0.37	0.65	-0.28
	UUG	1.01	1.17	-0.16	*	0.91	1.06	-0.15		1.27	1.29	-0.02
	CUU	1.21	1.39	-0.18		1.06	1.36	-0.30		1.41	1.47	-0.06
	CUC	1.30	1.03	+0.27	*	1.52	1.20	+0.32	*	1.72	1.13	+0.59
	CUA	0.51	0.58	-0.07		0.44	0.54	-0.10		0.47	0.64	-0.17
	CUG	1.65	1.33	+0.32	*	1.74	1.37	0.37		0.77	0.81	-0.04
Ile	AUU	1.00	1.18	-0.18		0.94	1.16	-0.22		1.01	1.09	-0.08
	AUC	1.41	1.06	+0.35	*	1.45	1.12	+0.33	*	1.60	1.14	+0.46
	AUA	0.59	0.76	-0.17		0.61	0.72	-0.11		0.39	0.77	-0.38
Val	GUU	1.03	1.33	-0.30		1.00	1.30	-0.30		1.42	1.47	-0.05
	GUC	1.09	1.01	+0.08	*	1.15	1.00	+0.15	*	1.03	0.83	+0.20
	GUA	0.44	0.53	-0.09		0.37	0.47	-0.10		0.34	0.54	-0.20
	GUG	1.44	1.13	+0.31	*	1.48	1.23	+0.25	*	1.21	1.16	+0.05
Ser	UCU	1.00	1.28	-0.28		0.96	1.28	-0.32		1.52	1.53	-0.01
	UCC	1.07	0.85	+0.22	*	1.13	1.00	+0.13	*	1.09	0.79	+0.30
	UCA	1.08	1.33	-0.25		1.06	1.21	-0.15		0.87	1.21	-0.34
	UCG	0.70	0.55	+0.15	*	0.84	0.59	+0.25		0.80	0.65	+0.15

	AGU	0.71	0.91	-0.20		0.67	0.84	-0.17		0.67	0.88	-0.21
	AGC	1.44	1.08	+0.36	*	1.33	1.08	+0.25	*	1.05	0.95	+0.10
Pro	CCU	1.20	1.34	-0.14		1.03	1.27	-0.24		1.54	1.49	+0.05
	CCC	0.80	0.62	+0.18	*	0.88	0.75	+0.13	*	0.64	0.54	+0.10
	CCA	1.13	1.46	-0.33		1.08	1.29	-0.21		1.01	1.26	-0.25
	CCG	0.86	0.58	+0.28	*	1.00	0.69	+0.31	*	0.80	0.70	+0.10
Thr	ACU	0.98	1.19	-0.21		0.87	1.12	-0.25		1.26	1.27	-0.01
	ACC	1.23	0.92	+0.31	*	1.31	1.03	+0.28	*	1.23	0.86	+0.37
	ACA	1.05	1.35	-0.30		0.99	1.28	-0.29		0.76	1.19	-0.43
	ACG	0.74	0.54	+0.20	*	0.84	0.57	+0.27	*	0.75	0.68	+0.07
Ala	GCU	1.20	1.42	-0.22		1.01	1.29	-0.28		1.72	1.70	+0.02
	GCC	1.13	0.88	+0.25	*	1.22	1.00	+0.22	*	0.80	0.64	+0.16
	GCA	0.98	1.17	-0.19		0.87	1.10	-0.23		0.72	1.05	-0.33
	GCG	0.69	0.52	+0.17	*	0.89	0.60	+0.29		0.76	0.61	+0.15
Tyr	UAU	0.75	0.94	-0.19		0.69	0.90	-0.21		0.65	0.89	-0.24
	UAC	1.25	1.06	+0.19	*	1.31	1.10	+0.21	*	1.35	1.11	+0.24
His	CAU	0.98	1.14	-0.16		0.88	1.08	-0.20		0.90	1.11	-0.21
	CAC	1.02	0.86	+0.16	*	1.12	0.92	+0.20	*	1.10	0.89	+0.21
Gln	CAA	0.68	0.83	-0.15		0.65	0.79	-0.14		0.82	0.99	-0.17
	CAG	1.32	1.17	+0.15	*	1.35	1.21	+0.14	*	1.18	1.01	+0.17
Asn	AAU	0.82	1.03	-0.21		0.82	1.01	-0.19		0.69	0.84	-0.15
	AAC	1.18	0.97	+0.21	*	1.18	0.99	+0.19	*	1.31	1.16	+0.15
Lys	AAA	0.59	0.79	-0.20		0.60	0.72	-0.12		0.64	0.91	-0.27
	AAG	1.41	1.21	+0.20	*	1.40	1.28	+0.12	*	1.36	1.09	+0.27
Asp	GAU	0.94	1.18	-0.24		0.92	1.12	-0.20		1.14	1.24	-0.10
	GAC	1.06	0.82	+0.24	*	1.08	0.88	+0.20	*	0.86	0.76	+0.10
Glu	GAA	0.71	0.91	-0.20		0.69	0.82	-0.13		0.77	0.93	-0.16
	GAG	1.29	1.09	+0.20	*	1.31	1.18	+0.13	*	1.23	1.07	+0.16
Cys	UGU	0.65	0.85	-0.20		0.64	0.81	-0.17		0.89	1.03	-0.14
	UGC	1.35	1.15	+0.20	*	1.36	1.19	+0.17	*	1.11	0.97	+0.14
Arg	CGU	0.72	0.79	-0.07		0.68	0.75	-0.07		1.17	0.95	+0.22
	CGC	0.99	0.85	+0.14	*	1.24	1.04	+0.20	*	0.77	0.48	+0.22
	CGA	0.48	0.60	-0.12		0.39	0.510	-0.12		0.55	0.58	-0.03
	CGG	0.93	0.75	+0.18		1.08	0.90	+0.18		0.42	0.52	-0.10

	AGA	1.03	1.29	-0.26		1.00	1.27	-0.27		1.44	2.01	-0.57
	AGG	1.85	1.73	+0.12	*	1.61	1.54	+0.07	*	1.65	1.45	+0.20
Gly	GGU	0.93	1.14	-0.21		0.83	1.06	-0.23		1.27	1.24	+0.03
	GGC	1.28	0.97	+0.31	*	1.46	1.17	+0.29	*	0.69	0.57	+0.12
	GGA	0.92	1.08	-0.16		0.80	0.98	-0.18		1.36	1.42	-0.06
	GGG	0.87	0.80	+0.07		0.91	0.79	+0.12		0.68	0.77	-0.09

[a] The total number of long genes and the average gene length, respectively, used to estimate *RSCU*: *Z. mays* sperm 476, 741.6 (±20.7), *Z. mays* egg 390, 655.4 (±18.9), *T. aestivum* anther 2093, 753.4 (±8.3), *T. aestivum* ovary 660, 661.7 (±13.0), *B. napus* microspore 582, 639.5 (±12.6), *B. napus* flower 1456, 676.1 (±8.6).

[b] Previously described as preferred codons in *Z. mays* and *T. aestivum* as indicated by their frequencies in high- versus low-biased genes by Kawabe and Miyashita [42]. Asterisk (*) indicates a preferred codon. No values available for *B. napus*.

Table 4. *P*-values for comparisons of *GC3* between short genes (less than or equal to 200 amino acids in the homologous *Arabidopsis thaliana* protein), medium length genes (more than 200 amino acids and less than or equal to 400) and long genes (more 400 amino acids) for female-specific and male-specific genes in *Zea mays* and *Triticum aestivum* and for male-specific and flower-specific genes in *Brassica napus*. Mean values are presented in Table 4 the main text. *P*-values are those for the Mann-Whitney Rank Sum Test (*t*-tests yielded similar *P*-values).

	Zea mays		*Triticum aestivum*		*Brassica napus*		
**Male**	Short	Medium	Short	Medium	**Male**	Short	Medium
Short					Short		
Medium	$<1.0X10^{-16}$		$<1.0X10^{-16}$		Medium	$<1.0X10^{-16}$	
Long	$<1.0X10^{-16}$	$<1.0X10^{-16}$	$<1.0X10^{-16}$	$<1.0X10^{-16}$	Long	$<1.0X10^{-16}$	$<1.0X10^{-16}$
**Female**					**Flower**		
Short					Short		
Medium	$<1.0X10^{-16}$		$<1.0X10^{-16}$		Medium	$<1.0X10^{-16}$	
Long	$<1.0X10^{-16}$	0.003	$<1.0X10^{-16}$	$<1.0X10^{-16}$	Long	$<1.0X10^{-16}$	$<1.0X10^{-16}$

Table 5. Mean GC content (at third codon positions) for female-specific and male-specific genes in *Zea mays* and *Triticum aestivum* and for male-specific and flower-specific genes in *Brassica napus* for each combination of gene expression level (low, high) and protein length (short, medium, long). Sample sizes were very small in some groups due to the separation of the gene datasets by both expression level and protein length (N ≤30) resulting in low power (low sample size, LSS). Mean protein lengths per category are provided in Table 3 of the main text. *P*-values are those for the Mann-Whitney Rank Sum Test (*t*-tests yielded similar *P*-values).

			Z ea mays		*Triticum aestivum*		*Brassica napus*		
			Mean (±SE)	*P*-value	Mean (±SE)	*P*-value		Mean (±SE)	*P*-value
Protein Length	Gene Expression								
**Short**									
	Low	Female	0.700 (0.013)		0.756 (0.012)		Male	0.528 (0.005)	
		Male	0.628 (0.016)	0.001	0.727 (0.011)	0.103 (LSS)	Flower	0.499 (0.005)	0.006
	High	Female	0.722 (0.033)		0.809 (0.019)		Male	0.522 (0.011)	
		Male	0.641 (0.227)	0.060 (LSS)	0.783 (0.042)	0.507 (LSS)	Flower	0.527 (0.025)	0.102 (LSS)
**Medium**									
	Low	Female	0.624 (0.011)		0.672 (0.009)		Male	0.488 (0.004)	
		Male	0.552 (0.010)	$1.0X10^{-16}$	0.616 (0.007)	$1.0X10^{-16}$	Flower	0.471 (0.003)	$1.0X10^{-16}$
	High	Female	0.749 (0.030)		0.727 (0.031)		Male	0.514 (0.012)	
		Male	0.489 (0.018)	$1.0X10^{-16}$	0.679 (0.051)	0.428 (LSS)	Flower	0.490 (0.026)	0.398 (LSS)

**Long**									
	Low	Female	0.580 (0.010)		0.609 (0.009)		Male	0.467 (0.004)	
		Male	0.480 (0.008)	$1.0X10^{-16}$	0.526 (0.004)	$1.0X10^{-16}$	Flower	0.448 (0.002)	$1.0X10^{-16}$
	High	Female	0.742 (0.033)		0.684 (0.040)		Male	0.498 (0.017)	
		Male	0.516 (0.028)	$1.0X10^{-16}$	0.525 (0.033)	0.002	Flower	0.439 (0.019)	0.040 (LSS)
